# Valorisation of the Inhibitory Potential of Fresh and Dried Fruit Extracts of *Prunus spinosa* L. towards Carbohydrate Hydrolysing Enzymes, Protein Glycation, Multiple Oxidants and Oxidative Stress-Induced Changes in Human Plasma Constituents

**DOI:** 10.3390/ph15101300

**Published:** 2022-10-21

**Authors:** Anna Magiera, Joanna Kołodziejczyk-Czepas, Karolina Skrobacz, Monika Ewa Czerwińska, Magdalena Rutkowska, Aleksandra Prokop, Piotr Michel, Monika Anna Olszewska

**Affiliations:** 1 Department of Pharmacognosy, Faculty of Pharmacy, Medical University of Lodz, 1 Muszynskiego St., 90-151 Lodz, Poland; 2 Department of General Biochemistry, Faculty of Biology and Environmental Protection, University of Lodz, 141/143 Pomorska, 90-236 Lodz, Poland; 3 Department of Biochemistry and Pharmacogenomics, Medical University of Warsaw, 1 Banacha St., 02-097 Warsaw, Poland; 4 Centre for Preclinical Research, Medical University of Warsaw, 1B Banacha St., 02-097 Warsaw, Poland; 5 Student Scientific Association, Department of Pharmacognosy, Faculty of Pharmacy, Medical University of Lodz, 1 Muszynskiego St., 90-151 Lodz, Poland

**Keywords:** *Prunus spinosa* Linne, polyphenols, antidiabetic activity, antioxidant activity, human plasma, digestive enzymes, glycation

## Abstract

*Prunus spinosa* fruits (sloes), both fresh and dried, are underexplored dietary components and ethno-phytotherapeutic remedies applied to treat chronic oxidative-stress-related diseases, including diabetes. The present study aimed to evaluate drying-related changes in the antidiabetic potential of sloe extracts and some bioactivity mechanisms, which might be connected with their traditional application. The polyphenol-enriched extracts, prepared by fractionated extraction and phytochemically standardised, i.a., by LC-MS/MS, were tested in vitro using a set of biological and chemical models. The experiments revealed the significant extracts’ ability to counteract the generation of advanced glycation end products (AGEs) and inhibit the activity of key glycolytic enzymes, i.e., α-glucosidase and α-amylase. Moreover, they were proved to effectively scavenge multiple oxidants of physiological importance (O_2_^•−^, HO^•^, H_2_O_2_, NO^•^, HOCl), increase the non-enzymatic antioxidant capacity of human plasma (NEAC) under oxidative stress conditions induced by peroxynitrite, and protect plasma proteins and lipids against peroxidation and nitration at in vivo-relevant levels (1–50 µg/mL, equivalent to 0.03–6.32 µg polyphenols/mL). In most cases, the activity of fresh fruit extracts surpassed that of dried-based products. The correlation studies and tests on model compounds proved polyphenols as dominant contributors to the observed effects. Furthermore, the co-occurring representatives of various polyphenolic classes were found to contribute to the biological activity of sloes through additive and synergistic effects. Considering the extraction yield and activity parameters, especially the superior outcomes compared to anti-diabetic drugs aminoguanidine and acarbose in the anti-glycation and α-glucosidase inhibition tests, the methanol–water (75:25, *v*/*v*) extract of fresh fruits and its phenolic-enriched fractions revealed the most advantageous potential for functional application.

## 1. Introduction

Diabetes mellitus (DM) is a chronic ailment distinguished by high blood-glucose levels and various vascular pathologies associated with hyperglycaemia, such as cardiomyopathy, peripheral arterial diseases, diabetic nephropathy or retinopathy [1]. Nowadays, the frequency of DM is increasing worldwide drastically because of the changing lifestyles and diets of industrial societies. The International Diabetes Federation expects that the diabetic population throughout the world will become almost 800 million by 2045, posing severe health and economic challenge [2].

Significant evidence from epidemiological studies has revealed that long-term consumption of plant-derived foods is inversely correlated with DM and its cardiovascular complications [3]. The beneficial health effects of plant products are primarily connected with their polyphenolic constituents, such as flavonoids, anthocyanins, phenolic acid derivatives, and proanthocyanidins. As therapeutic agents, polyphenols may act through various molecular mechanisms, including influence on the DM-related metabolic pathways, inhibition of protein glycation and generation of toxic end products of advanced glycation (AGEs), enhancement of the total antioxidant status, and modification of inflammatory response [3,4]. In addition, polyphenols can ameliorate hyperglycaemia by inhibiting the activity of critical enzymes digesting complex carbohydrates in the intestinal lumen, such as α-glucosidase and α-amylase [3,5]. Therefore, the development and biological effects of polyphenol-rich plant-based products that can prevent or treat DM and its complications has recently gained close attention [4].

*Prunus spinosa* L. (blackthorn, sloe), which belongs to the Rosaceae family, is a perennial deciduous plant growing native as a shrub in wild areas of northern Europe, Mediterranean countries, temperate regions of Asia and in northwest Africa. Moreover, as a naturalized species, the plant also occurs in the temperate zone of North America [6]. Blackthorn is a source of edible fruits—fresh plums are used to make syrups, juice, wine, liqueurs, and tinctures, while dried sloes are added to herbal teas [7]. The polyphenol-rich blackthorn fruits, both fresh and dried, are pleiotropic ethomedicines indicated to treat gastrointestinal and urinary inflammations, diarrhoea, and metabolic diseases, including diabetes and obesity [8,9]. Moreover, herbal formulations of fresh sloes are used as heart-strengthening and anti-hypertensive agents [10].

Although the ethnopharmacological sources suggested the potential of blackthorn fruits in the prophylaxis and therapy of DM and its cardiovascular pathologies [8,9,10], the relevant activity mechanisms and vectors are still insufficiently recognised. Most of the earlier works focused on the anti-inflammatory properties of the fruit preparations: the extracts were proved to downregulate the pro-inflammatory response of human immune cells, including neutrophils and peripheral blood mononuclear cells [11,12], and inhibit the expression of adhesion molecules in human endothelial cells [13], which might be essential for alleviating the DM-associated vascular inflammation. The potential direct antidiabetic properties of sloe fruits have been analysed to date only by Popović et al. [14]. They showed that alcoholic extracts of fresh sloes could inhibit α-glucosidase and α-amylase and investigated the impact of selected polyphenolic compounds on the observed activity. Unfortunately, the potential of dried fruits to inhibit glycolytic enzymes remained unexplored to date. On the other hand, plant extracts usually have a complex composition, and their biological capacities are driven by the joint activity of multiple constituents rather than by individual phytochemicals, with the benefit of additive and synergistic effects [15]. Our previous study revealed the vast complexity of the phenolic matrix of sloes; over 60 compounds representing various chemical classes were identified by LC-MS/MS, including flavonoids, anthocyanins, phenolic acids, tannin-type proanthocyanidins, and non-polyphenolic Maillard reaction products (MRPs), present only in the dried fruits [11,12]. Apart from the presence of MRPs, the extracts of fresh and dried *P. spinosa* fruits differed significantly in terms of the structure and concentration of numerous polyphenols [11,12]. Therefore, the contribution of various compounds/groups of compounds and differences in phenolic profiles of fresh and dried blackthorn fruits to their antidiabetic potential should be explained.

DM and DM-related vascular pathologies are linked to oxidative stress arising from the hyperglycaemia-induced excessive production of reactive oxygen and nitrogen species (ROS/RNS), which can trigger a maladaptive response by affecting numerous signalling and metabolic pathways in diabetic complications [1]. First, the oxidative steps are involved in glycation (glycoxidation), i.e., the non-enzymatic reaction between reducing sugars and protein amino groups, which leads to the generation of AGEs. They are metabolic pro-oxidants that can directly impair the functions of various proteins and further boost ROS/RNS secretion. The overproduced ROS/RNS can induce inflammatory reactions and attack various biomolecules in blood plasma, vascular endothelium, and myocardium, increasing tissue degradation [16]. Thus, the potential antiglycation effects of blackthorn fruits, their influence on functional biomolecules under oxidative stress conditions, or multiple ROS/RNS generated in vivo might represent important activity mechanisms of sloes in treating diabetes. Unfortunately, there are no previous studies on the subject to date.

Therefore, the purpose of the present study was: (a) to compare for the first time the inhibitory ability of the phenolic-enriched extracts from fresh and dried *P. spinosa* fruits (thoroughly standardised by various phytochemical methods, including LC-MS/MS) towards glycolytic enzymes (*α*-glucosidase and *α*-amylase); (b) to investigate the scavenging capacity of the extracts towards various ROS/RNS of physiological significance (O_2_^•−^, HO^•^, H_2_O_2_, NO^•^, HOCl); (c) to evaluate the anti-glycation properties of the extracts (the impact on the formation of AGEs); and (d) to verify the preventive features of the extracts against peroxidation and nitration of human plasma components, and their impact on the non-enzymatic antioxidant capacity of plasma under oxidative stress conditions. Moreover, correlation studies and comparative tests of native blackthorn polyphenols and MPRs were applied to specify compounds responsible for the effects observed in vitro. Finally, the extracts most favourable for their potential use as functional products were selected.

## 2. Results and Discussion

### 2.1. Inhibition of Digestive Enzymes Related to Diabetes Mellitus (DM)

The carbohydrate-metabolising enzymes α-glucosidase and α-amylase are among the leading pharmacological targets for managing DM [17]. The primary function of the two enzymes is to hydrolyse polysaccharides from food, specifically starch, to enable the absorption of free monosaccharides in the gastrointestinal tract. The breakdown of starch to maltose and other oligosaccharides is mainly catalysed in the duodenum by α-amylase synthesized by pancreatic acinar cells [18]. Subsequently, at the mucosal brush border of the small intestine cells, α-glucosidase hydrolyse the free oligosaccharides into single glucose units, which are then transported into the blood [19]. Therefore, inhibiting these enzymes slows down the digestion of complex sugars, reduces the absorption of the hydrolysis products, and consequently lowers the postprandial plasma sugar in DM patients.

Antioxidant-rich plant food has significant antidiabetic potential and is used to prevent and support the treatment of DM [20]. Fruits are among the best sources of bioavailable polyphenolic antioxidants in the human diet, with excellent potential for producing antidiabetic functional foods or even phytotherapeutics. The accumulated research revealed that the fruits of various *Prunus* species might be especially effective inhibitors of glycolytic enzymes related to DM [21,22,23]. Therefore, the present study aimed to compare the inhibitory ability of *P. spinosa* fresh and dried fruits towards both key carbohydrate-digesting enzymes α-glucosidase and α-amylase. The material for the study were the hydroalcoholic extracts of fresh (MEF) and dried (MED) sloes and their concentrated polyphenol-rich fractions obtained by fractionated extraction of the source extracts with diethyl ether (DEFs), ethyl acetate (EAFs), and *n*-butanol (BFs). Moreover, the post-extraction water residues (WRs) were also analysed to obtain a deeper insight into the activity vectors of the studied extracts. In our previous works, we showed that fractionation is a convenient way to establish active components of a studied plant because it enables selective isolation of various groups of phytochemicals and prevents the coincidental correlations that might be observed for extracts of similar composition [11,12,24].

**Figure 1:**
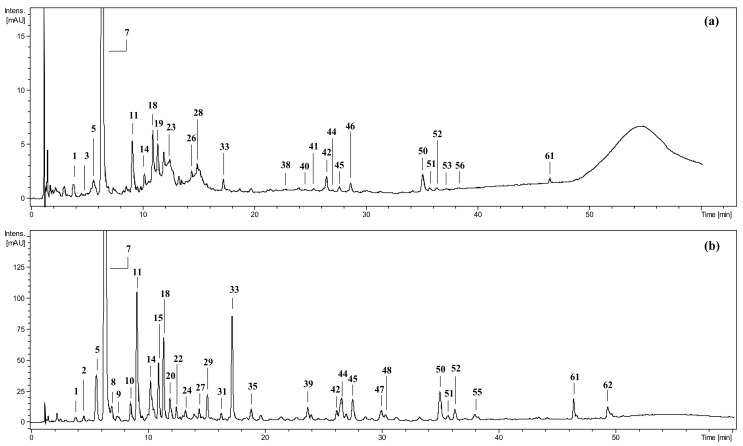
Example UHPLC chromatograms at 280 nm of (**a**) methanol–water (75:25, *v*/*v*) extract from fresh *P. spinosa* fruits, MEF; and (**b**) its ethyl acetate fraction, EAFF. Peak identification: 1—vanillic acid *O*-hexoside; 2—protocatechuic acid; 3—unidentified; 5—*cis*-3-*O*-caffeoylquinic acid; 7—neochlorogenic acid; 8—*p*-hydroxybenzoic acid; 9—neochlorogenic acid hexoside; 10—vanilloyl malate hexoside; 11—3-*O*-*p*-coumaroylquinic acid; 14—chlorogenic acid; 15—cis-3-*O*-feruloylquinic acid; 18—cryptochlorogenic acid; 19—cyanidin 3-*O*-glucoside; 20—caffeic acid 3/4-*O*-hexoside; 22—3-*O*-feruloylquinic acid; 23—cyanidin 3-*O*-rutinoside; 24—vanillin; 26—peonidin 3-*O*-glucoside; 27—*cis*-3-*O*-*p*-coumaroylquinic acid; 28—peonidin 3-*O*-rutinoside; 29—4-*O*-caffeoylshikimic acid; 31—4-*O*-feruloylquinic acid; 33, 35—caffeoylshikimic acid; 38—kaempferol dihexoside; 39—*p*-coumaroylshikimic acid; 40—quercetin hexoside-pentoside; 41—quercetin rhamnoside-hexoside; 42—hyperoside; 44—rutin; 45—isoquercitrin; 46—quercetin 3-*O*-(2″-*O*-*β*-D-glucopyranosyl)-*α*-L-arabinofuranoside; 47—reinutrin; 48—guaiaverin; 50—avicularin; 51—multinoside A; 52—quercitrin; 53—isorhamnetin rhamnoside-hexoside; 55—quercetin malyl-pentoside; 56—quercetin acetyl-hexoside; 61—quercetin acetyl-hexoside-rhamoside; 62—quercetin. Nomenclature of pseudodepsides according to IUPAC. For details of compounds’ identification and quantification see Magiera et al. [11].

The extracts/fractions for the present study were obtained previously, verified as non-cytotoxic for human blood cells in vitro, and thoroughly standardised using a panel of phytochemical techniques, including LC-MS/MS [11,12]. The standardisation allowed the identification and quantitation of 69 individual compounds as well as the calculation of total contents of flavonoids (TFL), phenolic acids (TPA), tannin-type proanthocyanidins (TTC), anthocyanins (TAC), and MRPs [11,12]. The representative chromatograms for fresh fruit extracts/fractions were provided in Figure 1. The overall phytochemical composition of the extracts/fractions was shown in Supplementary Table S1; as indicated, their total phenolic contents (TPC) fall in the range of 26.8–126.5 mg gallic acid equivalents (GAE)/g dw. The details on the structural identification and quantitative determination of polyphenolic individuals were reported in earlier works [11,12]. Among the identified compounds, cyanidin 3-*O*-glucoside, quercetin 3-*O*-glucoside (isoquercitrin), quercetin, chlorogenic acid (5-*O*-caffeoylquinic acid), and 5-hydroxymethylfurfural (HMF) were chosen as model representatives of various chemical classes to test the impact of individual phytochemicals on the analysed activity.

As presented in Table 1 and Supplementary Figure S1, most of the tested extracts/fractions acted as dose-dependent inhibitors of α-glucosidase and α-amylase but with different effectiveness towards a particular enzyme. The exceptions were WRF and WRD, which, up to a concentration of 1500 μg/mL, showed no activity against α-amylase. The estimated IC_50_ values indicated the extracts as more potent inhibitors of α-glucosidase than α-amylase, which follows the recent report of Popović et al. [14] on the ethanol–water extracts from fresh *P. spinosa* fruits from north Serbia. Moreover, the fresh fruit extracts were significantly more effective than those from dried sloes (Table 1 and Supplementary Figure S1).

The inhibitory activity of all analysed extracts/fractions towards α-glucosidase was superior compared to acarbose, a reference antidiabetic drug. The most active were hydroalcoholic fresh fruit extract (MEF) and DEFs from both fresh and dried sloes (Table 1 and Supplementary Figure S1). Their IC_50_ values amounted to 15.4–26.8 μg/mL and were up to 11.5 times lower than observed for acarbose (177.1 μg/mL), which means much stronger activity. However, the effect of all other extracts/fractions was also relevant and at least not worse than that of the drug (*p* < 0.05), regardless of whether they came from fresh or dried fruits. Although synthetic drugs, including acarbose, are widely recommended to treat DM and prediabetes, their medicinal application has numerous side effects, such as nausea, diarrhoea or flatulence [17]. Therefore, the proven effectiveness of sloe extracts suggests that both fresh and dried *P. spinosa* fruits are promising raw materials for producing antidiabetic functional foods with a preference for fresh ones.

It is worth noting that only a few fruit extracts have been demonstrated to inhibit α-glucosidase with similar effectiveness to the tested *P. spinosa* extracts/fractions. According to the latest literature, the comparable or more advantageous activity in relation to acarbose was achieved for alcoholic extracts from mulberry (*Morus alba* L.) [25], sea buckthorn (*Hippophae rhamnoides* L.) [26], pomegranate (*Punica granatum* L.) [27], and dogwoods (*Cornus* sp.) [28], while in the genus *Prunus* they were obtained for peach (*Prunus persica* (L.) Batsch) [21] and sweet cherry (*Prunus avium* L.) [23]. The reported IC_50_ values for these extracts amounted to 0.6–72.0 µg/mL, while the corresponding parameters of *P. spinosa* fruit extracts (the most active samples) tested in the present paper reached the values of 15.4–26.8 µg/mL. Therefore, it further supports the use of sloes and extracts thereof in the phytotherapy of DM, according to traditional recommendations [8,9].

**Figure 2:**
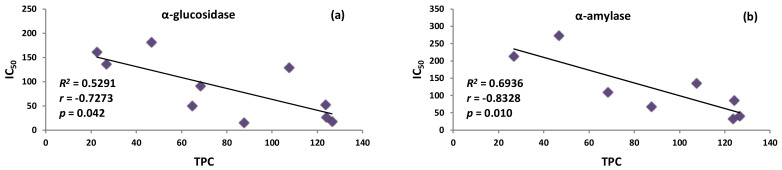
Correlation graphs between the total polyphenolic content (TPC) and: (**a**) α-glucosidase inhibition, (**b**) α-amylase inhibition of the extracts. Graphs plotted with the mean value of IC_50_ (μg/mL) vs. the mean value of TPC (mg GAE/g dw); *R*_2_, coefficient of determination; *r*, correlation coefficients; *p*, probability values.

As shown in Table 1 and Supplementary Figure S1, fresh fruit extract (MEF) demonstrated approximately nine times stronger inhibitory activity towards α-glucosidase than the dried fruit extract (MED): their IC_50_ values were 15.4 μg/mL and 136.3 μg/mL, respectively. At the same time, MEF was characterised by much higher total contents of polyphenols (TPC and TPH) and polyphenolic subgroups (*p* < 0.05), especially tannin-type proanthocyanidins (TTC); additionally, MEF was distinguished by the presence of anthocyanins (TAC), not detected in MED (for details on the drying-related changes in the *P. spinosa* phytochemistry see Magiera et al. [12]). This might suggest that proanthocyanidins and anthocyanins might be primarily responsible for α-glucosidase inhibition. This assumption was supported by the results obtained for the BFs and WRs fractions—those from fresh fruits accumulated significantly higher TAC and TTC levels than those from dried sloes (*p* < 0.05). However, the DEFs and EAFs clearly broke out of the pattern as they did not contain either tannins or anthocyanins; instead, they accumulated elevated levels of phenolic acids (TPA) and flavonoids (TFL). It strongly suggested that none of the phenolic groups or individual compounds alone but all polyphenols of the fruits contribute to the observed effects by synergistic or additive mechanisms. The relatively low effectiveness of pure model polyphenols, especially cyanidin 3-*O*-glucoside and chlorogenic acid (Table 1 and Supplementary Figure S1), and the statistically significant correlation between the TPC levels and activity parameters for α-glucosidase inhibition (*r* = −0.7273, *p* < 0.05; Figure 2a, Supplementary Table S2), confirmed this hypothesis. Our results thus partially contrast those of Popović et al. [14], who observed only a relevant correlation of the α-glucosidase inhibitory activity with the levels of quercetin 3-*O*-glucoside (isoquercitrin) in the sloe extract without, however, testing the actual potential of the pure compound. As shown in Table 1 and Supplementary Figure S1, the reactivity of isoquercitrin cannot solely explain the activity of *P. spinosa* extracts. On the other hand, the relatively high IC_50_ values of polyphenolic standards might suggest that some non-phenolic compounds may also influence the inhibitory capacity of the extracts. Previously, Cui et al. [22] reported on the potent inhibitory capacity of a polysaccharide fraction from the apricot pulp (*Prunus armeniaca* L.) towards α-glucosidase. Therefore, further research on blackthorn fruits should address the possible influence of non-phenolic phytochemicals on their anti-glycolytic activity.

The synergic contribution of polyphenols was also proved for the α-amylase inhibition through a close correlation with the TPC values (*r* = −0.8328, *p* < 0.01; Figure 2b, Supplementary Table S2). This time, polyphenols appeared to be the essential vectors of the observed effects as the model polyphenolic compounds revealed the relevant activity with the most decisive impact of flavonoids and anthocyanins (quercetin, cyanidin 3-*O*-glucoside). It confirmed the report of Popović et al. [14] on a similarly strong relationship between 1/IC_50_ values for α-amylase inhibition and TPC levels (*r* = −0.8085, *p* < 0.01), as well as some individual quercetin glycosides and anthocyanins in the fresh *P. spinosa* fruit extracts. Likewise, the activity parameters of sloe extracts were worse than that found for acarbose in the present (Table 1 and Supplementary Figure S1) and earlier study [14]. We observed that the most potent extracts/fraction from fresh fruits (MEF, DEFF, EAFF) inhibited the enzyme 7–14 times weaker than the reference drug, while the capacity of dried fruit extracts/fraction was even lower. Therefore, only the fresh fruits might be considered potential α-amylase inhibitors, and the concentrated fruit fractions seem to be the most valuable for the production of functional food. The enrichment in polyphenols is more recently becoming an increasingly popular procedure when preparing functional products [29].

The results obtained in the inhibition tests towards glycolytic enzymes may also provide a preliminary explanation of the antidiabetic activity mechanisms of sloe fruits. Our results show the selectivity of the extracts in terms of activity mechanisms—they are more potent inhibitors of α-glucosidase than α-amylase. It should be noted that such selectivity is beneficial for a functional product candidate and is preferred by modern medicine and pharmacy. Furthermore, such selectivity is often observed for polyphenol-rich plant extracts and is probably related to the differences in specific steric affinity of polyphenols to both these enzymes. For instance, such variations in the inhibitory potential for a given enzyme might be related to the number of hydroxyl groups and the formation of hydrogen bonds between the hydroxyl groups of polyphenolic compounds and the catalytic residues of the enzyme binding sites [23].

### 2.2. Scavenging of Multiple ROS

ROS/RNS are generated as by-products of the normal oxygen metabolism and have an essential regulatory role for anti-microbial defense and various cellular functions. However, the hyperglycemic conditions in DM can lead to the overproduction of ROS/RNS and the development of systemic oxidative stress. Among different ROS, the superoxide anion radical (O_2_^•−^) is critical in vivo because it is the primary species generated by immune cells upon different pro-oxidant and pro-inflammatory stimuli. When produced, O_2_^•−^ is transformed into other toxic ROS/RNS, including hydroxyl radical (HO^•^), hydrogen peroxide (H_2_O_2_), nitrogen oxide (NO^•^), hypochlorous acid (HOCl), and peroxynitrite (ONOO^−^) [30]. The increased ROS/RNS production modifies numerous DM-related pathways implicated in the pathogenesis of diabetic complications, such as the polyol and hexosamine pathways, nuclear factor kappa B (NF-κB) signalling pathway, as well as the generation of AGEs and protein kinase C activation [16,31]. Therefore, the supportive role of dietary polyphenols in the treatment of DM is often explained by their antioxidant effects and powerful ability to neutralise a ROS/RNS family in the mitochondrial electron transport chain. The polyphenolic antioxidants disturb the oxidative sequence in different ways; they may intercept singlet oxygen, prevent the first-chain initiation by scavenging O_2_^•−^, decompose primary oxidation products to non-radical species, bind metal ion catalysts, and interact with various essential signalling proteins [32].

This study explored the scavenging ability of blackthorn fruits towards in vivo-relevant ROS/RNS for the first time. Previously, only their impact on synthetic free-radicals, such as DPPH or ABTS^•+^, was mainly investigated [33,34]. As indicated in Table 2 and Supplementary Figure S2, all of the analysed extracts/fractions of *P. spinosa* fruits showed a significant and concentration-dependent capacity to scavenge five of the most common ROS/RNS generated in vivo: O_2_^•−^, HO^•^, H_2_O_2_, NO^•^, and HOCl. The especially low SC_50_ values, compared to the positive controls of ascorbic acid and Trolox, were found in the NO^•^, HOCl, and O_2_^•−^-scavenging tests. In all tests, MEF revealed more potent effects than MED, indicating stronger antioxidant activity of fresh fruits in relation to dried sloes. Nevertheless, the SC_50_ values expressed in μg GAE/mL for all fractions (re-calculated from the original data with the TPC levels; Supplementary Table S3) showed that blackthorn polyphenols of both fresh and dried fruits are effective even at very low concentrations. For example, in the O_2_^•−^-scavenging test, the re-calculated values were 2.7–7.0 µg GAE/mL, while the respective parameters for model polyphenols and positive controls amounted 5.3–135.3 µg/mL. Among all extracts/fractions, the most potent effects were found for DEFs and EAFs. It corresponded to our previous reports on the powerful antioxidant effects of these fractions in human neutrophils ex vivo: at physiological concentrations (2.5–5 µg/mL; 0.3–0.6 µg GAE/mL), they significantly limited the ROS secretion in the *f*-MLP-stimulated cells by approximately 36–69% [11,12]. The present results might thus suggest that the cellular antioxidant effects of sloes are partly due to their direct ROS-neutralising potential. Likewise, Fraternale et al. [35] observed that sloe juice increases the viability of H_2_O_2_-stressed premonocytes, probably due to H_2_O_2_-scavenging. On the other hand, Sabatini et al. [13] have documented that the target activity is also connected with the blockage of the NF-κB signalling.

The correlation studies (Supplementary Table S2) and tests on model compounds (Table 2) proved that polyphenols significantly contribute to the observed scavenging effects. For all scavenging assays, the essential impact (*p* < 0.05) of total phenolic contents (TPC, *r* > −0.88; TPH, *r* > −0.71) was evidenced, and O_2_^•−^-scavenging also significantly depended (*p* < 0.05) on the total flavonoid content (TFL, *r* > −0.79). Similarly, as in the digestive enzyme inhibition tests, it suggested a strong synergy between individual blackthorn polyphenols. Many scientific reports point to the antioxidant synergy of polyphenols. For example, the synergism of chlorogenic acid with quercetin, rutin, isoquercitrin, and a quercetin pentoside-hexoside has previously been documented [15,36,37].

### 2.3. Inhibition of the AGEs Generation

The overproduction of ROS in hyperglycemic conditions also accelerates the protein glycation and formation of AGEs. By binding with each other and with proteins, AGEs form a network of cross-links and disturb the functions of most cells and tissues in the human body [38]. For example, the glycation of collagen in the vascular wall makes its fibres stiff and inelastic, which results in the loss of elasticity of blood vessels, a typical complication of DM. The breakdown of endothelial cells from the extracellular matrix is also attributed to protein glycation. This may favour the development of atherosclerotic plaque and diabetic microangiopathies. In addition, the glycation of lipoproteins of cell membranes increases their permeability, facilitates lipid peroxidation, and causes oxidative damage to intracellular structures. Furthermore, the binding of AGEs to the AGEs-specific receptor (RAGE) activates immune cells, such as monocytes/macrophages, granulocytes, and T lymphocytes, leading to the development of chronic inflammation [38]. Recently, it has been suggested that AGEs may also damage pancreatic β-cells and contribute to insulin resistance [39]. Dietary polyphenols are among the most promising natural anti-glycation agents [40,41]. According to the available data, the anti-glycation activity of polyphenols is connected with various direct and indirect mechanisms, such as ROS-scavenging, trapping reactive dicarbonyl compounds, deactivating carbonyl groups of reducing monosaccharides, binding with proteins, cleaving the AGEs structures, and downregulating the RAGE expression [41,42].

In this study, the polyphenol-rich *P. spinosa* fruits were, for the first time, demonstrated to counteract non-enzymatic protein glycation (Table 1 and Supplementary Figure S1). All of the tested extracts/fractions prevented the formation of AGEs with effectiveness comparable to or higher than the reference aminoguanidine. Although it is a prototype anti-AGEs agent approved for treating some diabetic complications, it has severe side effects; thus, other glycation inhibitors are in demand [40]. In this context, the most promising blackthorn extracts are MEF, BFF, and DEFD, revealing significantly stronger anti-glycation effects than aminoguanidine (*p* < 0.05). As shown in Table S1, MEF and BFF were rich in anthocyanins and proanthocyanidins. However, the correlation tests (Supplementary Table S2) displayed only a moderate relationship between the anti-AGEs capacity of the extracts and the levels of these groups of polyphenols (*r* = −0.6820 for TAC and *r* = −0.5288 for TTC; *p* > 0.05). Moreover, the experiments on model compounds (Table 1 and Supplementary Figure S1) suggested the relevant impact of flavonoids and phenolic acids, which might be essential for the elevated effects of DEFD. Therefore, again, the additive or synergistic effects of various polyphenols might better explain the activity of *P. spinosa* extracts. Previously, similar conclusions were drawn for anthocyanin-rich and anthocyanin-free extracts of blackberries, raspberries, blueberries, cranberries, and strawberries [43]. Moreover, our results also show that the sloe fruit extracts are more effective inhibitors of AGEs formation than those of many other fruits, commonly consumed or used in phytotherapy. The IC_50_ values of the basic hydroalcoholic extracts of sloes are in the range of 42.9–96.8 μg/mL, while the methanol extracts of blackberries, black/red raspberries, and strawberries inhibited protein glycation by only 40% at 100 μg/mL. Lower antiglycation effectiveness was also observed for extracts of various *Cotoneaster* fruits (IC_50_ = 106.4–166.6 μg/mL) [44]. On the other hand, effects comparable to blackthorn extracts were obtained for the extracts of blueberries and cranberries [43].

### 2.4. Antioxidant Protection of Human Plasma Components against Oxidative Stress

Oxidative stress is a key factor in the pathogenesis of diabetes complications, especially within the cardiovascular system [31]. Apart from influencing numerous metabolic pathways and cell signalling and promoting the formation of AGEs, the overproduced ROS/RNS have a direct destructive effect on various biomolecules. One of the most toxic ROS/RNS generated under hyperglycemic conditions is peroxynitrite (ONOO^−^), produced in vivo by the reaction between O_2_^•−^ and NO^•^ radicals [45,46]. Once generated, ONOO^−^ induces structural modifications of functional proteins and lipids in the vascular endothelium and blood plasma, including nitration and dimerisation of tyrosine units, oxidation of active thiol groups in sulphur-containing amino acids, especially cysteine, and lipid peroxidation, which eventually leads to chronic cardiovascular dysfunction [47].

Therefore, to check the potential preventive effects of *P. spinosa* fruit extracts against oxidative and nitrative injury of biomolecules, an experimental model of human blood plasma exposed to oxidative stress induced by ONOO^−^ (100–150 μM; achievable locally in vivo, e.g., in inflamed blood vessels) was applied [46]. The process was monitored by measuring the oxidative stress biomarkers, including the products of protein nitration (3-nitrotyrosine, 3-NT), lipid peroxidation (thiobarbituric acid reactive substances, TBARS), as well as the total antioxidant capacity of plasma (NEAC). The extracts and fractions from fresh sloes were selected for the study due to their high ability to inhibit glycolytic enzymes, potent anti-glycation activity, and significant ROS/RNS-scavenging properties. In addition, the basic hydroalcoholic extract of dried fruits (MED) was tested for the overall comparison. The extracts/fractions were assayed at 1–50 µg/mL concentrations, equal to 0.03–6.32 µg GAE/mL, depending on the TPC value. As discussed earlier with the accumulated bioavailability data, dietary polyphenols may reach such levels in plasma after oral supplementation [24,47].

**Figure 3:**
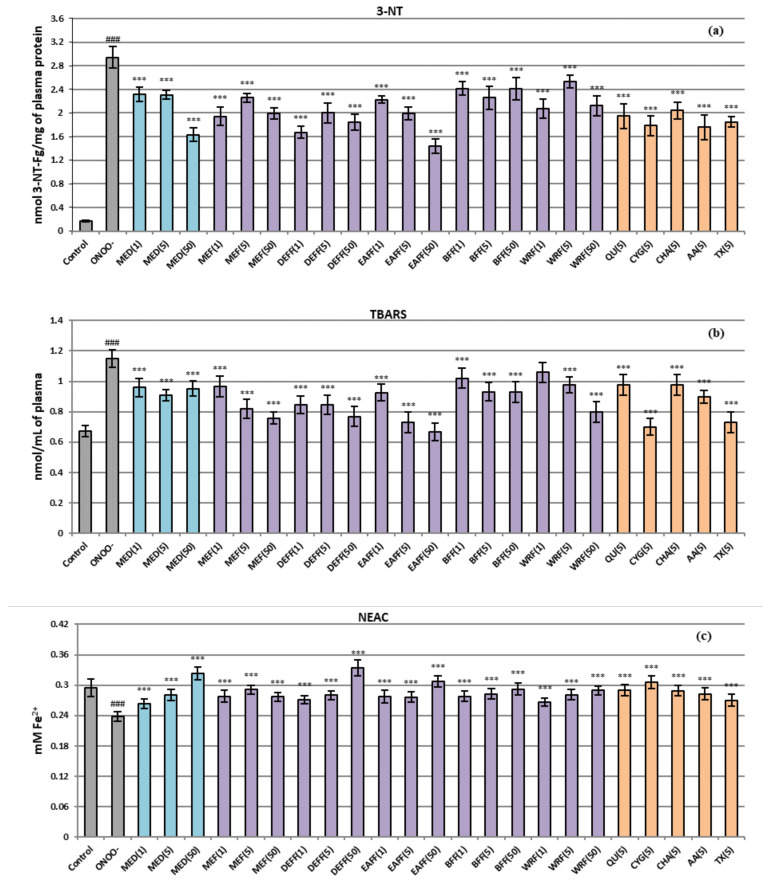
Protective effects of *P. spinosa* fruits extracts on human plasma under oxidative stress conditions: (**a**) influence on the nitration of tyrosine residues in plasma proteins and formation of 3-NT; (**b**) impact on the lipid peroxidation and TBARS generation; (**c**) effects on the NEAC value of plasma (assessed by FRAP). Data expressed as mean values ± SE (*n* = 7–9). Statistical significance: ### *p* < 0.001 for control plasma versus ONOO^−^-treated plasma in the absence of the investigated analytes; *** *p* < 0.001 for ONOO^−^-treated plasma in the presence of the extracts (1–50 μg/mL) or model compounds/positive controls (5 μg/mL) versus ONOO^−^-treated plasma without the analytes. QU, quercetin; CYG, cyanidin 3-*O*-glucoside; CHA, chlorogenic acid; AA, ascorbic acid; TX, Trolox^®^. Protective effects of *P. spinosa* fruits extracts on human plasma under oxidative stress conditions: (**a**) influence on the nitration of tyrosine residues in plasma proteins and formation of 3-NT; (**b**) impact on the lipid peroxidation and TBARS generation; (**c**) effects on the NEAC value of plasma (assessed by FRAP). Data expressed as mean values ± SE (*n* = 7–9). Statistical significance: ### *p* < 0.001 for control plasma versus ONOO^−^-treated plasma in the absence of the investigated analytes; *** *p* < 0.001 for ONOO^−^-treated plasma in the presence of the extracts (1–50 μg/mL) or model compounds/positive controls (5 μg/mL) versus ONOO^−^-treated plasma without the analytes. QU, quercetin; CYG, cyanidin 3-*O*-glucoside; CHA, chlorogenic acid; AA, ascorbic acid; TX, Trolox^®^.

As shown in Figure 3, the ONOO^−^-stimulated plasma exhibited considerably enhanced (*p* < 0.001) amounts of 3-NT and TBARS (an approximately 17-fold and 2-fold increase was observed, respectively) compared to the untreated samples. Moreover, a pronounced decrease in the NEAC value was revealed. In plasma samples co-incubated with ONOO^−^ and the extracts/fractions, the nitration and oxidation of both proteins and lipids were significantly limited (Figure 3a,b), regardless of the type and concentration of the tested extracts. Even at low levels of 1–5 μg/mL (0.02–0.63 µg GAE/mL), the fresh fruit extracts/fractions lowered tyrosine nitration by about 23–43% (DEFF and MEF were the most potent) (Figure 3b). At 50 μg/mL (3.23–6.32 µg GAE/mL), the 3-NT level was reduced by up to 51% for EAFF. The dried fruit extract MED was slightly less active; it reduced the nitration effect by about 22% at 1–5 μg/mL (0.01–0.13 µg GAE/mL) and 44% at 50 μg/mL (1.34 µg GAE/mL).

All tested fresh fruit extracts (except WRF at 1 μg/mL) also protected plasma against ONOO^−^-induced lipid peroxidation (Figure 3b), regardless of the concentration level. The most pronounced effect was observed at 5–50 μg/mL; in those conditions, the TBARS level for the samples incubated with EAFF, DEFF, and MEF was not significantly different (*p* > 0.001) or only 1.2-fold higher than that of untreated, control plasma. At 5–50 μg/mL, all analysed extracts/fractions also restored the NEAC plasma values to the physiological control levels (Figure 3c). Moreover, the highest enhancement of the NEAC values (to about 130–140% vs. the ONOO^−^-stimulated plasma) was found at 50 μg/mL of DEFF, EAFF and MED.

Generally, the fresh fruit extracts/fractions, featured by the highest phenolic contents (DEFF, EAFF, MEF), especially those enriched with flavonoids and phenolic acids (DEFF, EAFF), were the most valuable plasma protecting agents. The significant impact of polyphenols on the revealed effects was verified by the strong correlation (*r* = −0.83, *p* < 0.001) between the TPC and TBARS levels. For other tests, where the concentration-dependency was visible for selected extracts/fractions, the correlations were insignificant (*p* > 0.001), and the contribution of polyphenols was confirmed by the studies of model compounds. It was revealed that the preventive effects of quercetin, cyanidin 3-*O*-glucoside, and chlorogenic acid at 5 μg/mL did not differ substantially (*p* > 0.001) from that of positive standards ascorbic acid and Trolox (Figure 3), tested at the same concentration. Moreover, the standard polyphenols did not differ (*p* > 0.001) in antioxidant effectiveness from each other, except that cyanidin 3-*O*-glucoside was especially active in the TBARS test. All these observations suggest the synergistic/additive effects of various groups of polyphenols in the examined extracts. Similar effects were previously observed for some other fruits recommended by ethnomedicine for the adjunctive treatment of diabetes, such as rowanberries [24], and for blackthorn flowers [48]. On the other hand, the more potent capacity of the flower extracts observed previously and related to the higher phenolic contents in flowers [48] compared to the currently analysed fruits confirmed the significant contribution of polyphenols to the protective activity of blackthorn extracts in plasma.

According to the available research, the elevated levels of the investigated oxidative stress biomarkers and reduced plasma antioxidant capacity are valuable factors for predicting cardiovascular events [49]. Therefore, as the impact of exogenous non-enzymatic antioxidants on the plasma redox status in vitro corresponds with their in vivo effects [50], the reported activity of *P. spinosa* fruit extracts at low concentration levels might be an argument for their use as a functional food adjuvant in DM. However, further studies are required to verify their value in vivo experimentally.

### 2.5. Biological Potential of Maillard Reaction Products (MRPs) in Dried Fruits

Our previous study pointed to the presence of several MRPs in dried *P. spinosa* fruits [12]. These are drying-related constituents formed at elevated temperatures in a multistep reaction of amino acids and reducing carbohydrates [51]. The primary MPR in sloes is 5-hydroxymethylfurfural (HMF), present at the levels of 0.26–37.8 mg/g dw in the extracts/fractions of dried fruits (Table S1). HMF is also one of the most commonly occurring MPRs in food [51,52]. According to the available literature, HMF may have various biological effects, either beneficial or harmful. The molecular structure of HMF and the presence of some reactive functional groups, such as an aldehyde group, heterocyclic furan ring, and conjugated double bonds, have been suggested as the basis for the expected antioxidant activity of HMF [51,52]. However, in our previous research on the ROS-release in human neutrophils, the compound revealed relatively low activity; it downregulated the ROS level by only 15% at 50 μM, compared to a 96% reduction observed for quercetin at the same concentration [12]. The present study proved that HMF up to 500–1500 μg/mL (equivalent to 4–40 μM) has no direct ROS-scavenging capacity (Table 2 and Supplementary Figure S2). Moreover, it is inactive in the inhibition tests towards glycolytic enzymes (Table 1 and Supplementary Figure S1) and unable to inhibit the generation of AGEs (Table 1 and Supplementary Figure S1).

## 3. Materials and Methods

### 3.1. Study Material, Extraction and Phytochemical Standardisation

The research material was the extracts and fractions of fresh and dried *P. spinosa* L. fruits obtained by fractionated extraction as described previously [11,12]. The ripe fruits for the study were harvested in October 2018 from the natural habitats in Krasnobród (Poland) and divided into two stock samples: one immediately frozen to preserve fresh fruits and second dried as described earlier [11,12]. Eventually, five various extracts/fractions were prepared from each material. Briefly, methanol–water (75:25, *v*/*v*) extract (MEF) and its fractions of diethyl ether (DEFF), ethyl acetate (EAFF), *n*-butanol (BFF), and water residue (WRF) were produced from fresh fruits. Likewise, dried fruits yielded the source extract MED and its DEFD, EAFD, BFD, and WRD fractions. Details on the plant material origin, its authentication, storage conditions, voucher specimen number, the preparation of extracts/fractions, and the evaluation of their cellular safety in vitro, as well as on their comprehensive phytochemical standardisation using a set of chromatographic and spectrophotometric methods, including LC-MS/MS, were reported in the previous papers [11,12].

### 3.2. α-Glucosidase and α-Amylase Inhibition Assays

The inhibition of α-glucosidase was evaluated according to the spectrophotometric method reported by Ma et al. [53] with slight modifications. The enzyme activity measurement was based on the *p*-nitrophenyl-*α*-D-glucopyranoside (*p*-NPG) hydrolysis. In brief, 150 µL of a phosphate buffer (0.1 M, pH 6.9) solution of an extract or standard was mixed with the enzyme (0.43 U/mL, 50 µL) and pre-incubated for 15 min at 37 °C. Next, a 0.7 mM *p*-NPG solution in the buffer (50 µL) was added, and after 15 min of incubation, the reaction was stopped by adding Na_2_CO_3_ (0.2 M, 50 µL). Finally, the absorbance was read at 405 nm in a microplate reader. The positive control was acarbose. The values obtained for the blind samples without the analytes or acarbose meant 100% enzyme activity. The inhibitory potential of the analytes towards *α*-glucosidase was expressed as a half inhibitory concentration (IC_50_), calculated from concentration-inhibition curves.

The inhibitory activity of the analytes towards α-amylase was determined fluorometrically according to Truba et al. [28] using an EnzChek™ Ultra Amylase Assay Kit. The test was based on measuring products formed after the enzymatically catalysed hydrolysis of the corn starch substrate DQTM (labeled with the fluorescent dye BODIPY^®^ FL). The fluorescence of the hydrolysate was read at 37 °C in a microplate reader at 485 nm and 535 nm for excitation and emission, respectively. The positive control was acarbose. The values obtained for the blind samples without the analytes or acarbose meant 100% enzyme activity. The inhibitory potential of the analytes towards *α*-amylase was expressed as a half inhibitory concentration (IC_50_), calculated from concentration-inhibition curves.

### 3.3. Multiple Oxidants Scavenging Activity

The scavenging efficiency of *P. spinosa* fruit extracts/fractions towards various ROS (O_2_^•−^, HO^•^, H_2_O_2_, HOCl, and NO^•^) was tested using appropriate fluorimetric and spectrophotometric methods as described previously [54]. Ascorbic acid and Trolox^®^ served as positive controls. The results were expressed as half scavenging concentration (SC_50_) values, estimated using concentration-scavenging curves.

### 3.4. Inhibition of the Formation of Advanced Glycation Endproducts (AGEs)

The impact of the extracts/fractions on protein glycation was evaluated in the fructose-bovine serum albumin (BSA) test system with aminoguanidine as a positive control according to the fluorimetric method of Starowicz and Zieliński [42], modified by Rutkowska et al. [24]. The fluorescence was measured at 37 °C in a microplate reader at 355 nm and 420 nm for excitation and emission, respectively. The results were reported as half inhibitory concentration (IC_50_) values, estimated using concentration-inhibition curves.

### 3.5. Protective Effects on Human Plasma Exposed to Oxidative Stress

Blood plasma was isolated by differential centrifugation [48,55] of buffy coats (from healthy volunteers) obtained from the Regional Centre of Blood Donation and Blood Treatment in Lodz (Poland). The Committee on the Ethics of Research at the University of Lodz approved all experiments (decision number: 8/KBBN-UŁ/II/2015). For the study, plasma samples were pre-incubated for 15 min at 37 °C with the extracts/fractions (1–50 μg/mL) or model polyphenols/positive controls (Trolox^®^, ascorbic acid) and then treated by 100–150 μM ONOO^−^. Two kinds of control plasma were also prepared; the first was exposed to ONOO^−^ in the absence of the analytes, and the second was the blind control containing neither analytes nor ONOO^−^. Several experiments conducted with blood plasma and the analytes only (without ONOO^−^) confirmed the lack of their direct interactions with plasma components. The influence on the levels of oxidative stress biomarkers in plasma was evaluated using the appropriate immunoenzymatic and spectrophotometric methods [48,55]. In brief, a competitive ELISA test was applied to immunodetect 3-NT in proteins. The results were calculated using the standard curve of nitro-fibrinogen (3-NT-Fg) and expressed as 3-NT-Fg equivalents (nmol/mg of plasma proteins). The TBARS levels of plasma lipids were determined spectrophotometrically and calculated in nmol/mL of plasma. The impact on the NEAC value was measured using the FRAP assay, with the results calculated from the standard curve of ferrous sulphate and expressed in mM of Fe^2+^ equivalents [48,55].

### 3.6. Statistical Analysis

Statistical tests were done using the Satistica13Pl software for Windows (StatSoft Inc., Krakow, Poland). All results were presented as means ± SD (standard deviation) or ±SE (standard error) for the indicated number of independent replicates. Statistical comparisons were made using the parametric method of one-way ANOVA (for chemical tests) or one-way ANOVA for repeated measures (for human plasma model), followed by the post hoc Tukey’s test or Dunnett’s test. Pearson correlations were used to evaluate the relationships between the various parameters. Differences between the mean values and correlation coefficients were considered significant at *p*-values less than 0.05.

## 4. Conclusions

The present paper is the first investigation of the potential of polyphenol-rich extracts from fresh and dried *P. spinosa* fruits in the context of diabetes and its complications. Both fresh and dried fruit extracts revealed high antioxidant capacity, manifested in the potent direct scavenging of multiple oxidants generated in vivo, effective prevention against the generation of AGEs, enhancement of the non-enzymatic capacity of plasma under oxidative stress conditions, and noticeable protection against the nitrative and oxidative structural changes in plasma proteins and lipids. Moreover, the extracts were confirmed as powerful inhibitors of glycolytic enzymes, especially α-glucosidase. Polyphenolic compounds seem to contribute mainly to the observed effects through additive and synergic action. In most tests, the fresh fruit extracts exhibited more potent effects than those obtained from dried sloes. It confirmed that the changes in fruit polyphenols during drying have substantial deteriorative consequences on the biological capacity of the final products. The obtained results might partly explain the therapeutic application of fresh and dried *P. spinosa* fruits in diabetes, recommended by traditional medicine, but suggest that the fresh fruit should be favoured. Considering its high extraction yield and promising activity parameters compared to antidiabetic drugs and reference antioxidants, the hydroalcoholic extract of fresh sloes (MEF) appears to be most advantageous for biological application. However, additional research is demanded to confirm its effects in vivo and obtain a deeper insight into other possible mechanisms related to the treatment of diabetes and its complications. Moreover, the impact of non-phenolic constituents, including polysaccharides, on fruit activity should be addressed in future studies.

## Figures and Tables

**Table 1:** Inhibitory features of *P. spinosa* fruit extracts against glycolytic enzymes and protein glycation.

	α-Glucosidase IC_50_ (µg/mL)	α-Amylase IC_50_ (µg/mL)	AGEs Formation IC_50_ (µg/mL)
**Analytes:**			
MEF	**15.43 ± 0.84 ^A^**	68.32 ± 2.62 ^C^	**42.94 ± 3.18 ^B^**
MED	136.31 ± 4.24 ^D^	214.13 ± 12.20 ^F^	96.83 ± 3.41 ^D^
DEFF	**18.11 ± 0.42 ^A^**	**40.89 ± 2.04 ^B^**	73.61 ± 3.09 ^C^
DEFD	**26.82 ± 2.75 ^A^**	86.45 ± 3.71 ^C^	**51.51 ± 3.57 ^B^**
EAFF	52.39 ± 2.30 ^B^	**33.47 ± 1.57 ^B^**	75.34 ± 4.42 ^C^
EAFD	128.81 ± 6.29 ^D^	136.17 ± 10.18 ^E^	79.79 ± 5.39 ^C^
BFF	90.95 ± 13.00 ^C^	110.12 ± 15.46 ^D^	**45.50 ± 3.03 ^B^**
BFD	181.64 ± 9.23 ^F^	272.83 ± 4.18 ^G^	95.53 ± 2.67 ^D^
WRF	50.05 ± 2.10 ^B^	***	82.93 ± 2.07 ^C^
WRD	161.06 ± 3.52 ^E^	***	97.97 ± 5.62 ^D^
Quercetin	*	37.52 ± 2.32 ^B^	-
Quercetin 3-*O*-glucoside (isoquercitrin)	209.64 ± 5.64 ^G^	-	3.09 ± 0.17 ^A^
Cyanidin 3-*O*-glucoside	635.13 ± 34.43 ^H^	31.28 ± 1.66 ^B^	10.90 ± 0.85 ^A^
Chlorogenic acid	**	81.06 ± 3.94 ^C^	7.07 ± 0.74 ^A^
5-Hydroxymethylfurfural (HMF)	**	**	**
**Reference standards:**			
Acarbose	177.07 ± 9.10 ^E,F^	4.90 ± 0.30 ^A^	-
Aminoguanidine	-	-	78.89 ± 4.95 ^C^

IC_50_, Half inhibitory concentration (amount needed for 50% inhibition of a given process) expressed in µg/mg of the dry extract or standard/mL of the reaction mixture; * inactive up to a concentration of 50 µg/mL, above 50 µg/mL insoluble; ** inactive up to 1000 µg/mL; *** inactive up to 1500 µg/mL. For extracts codes, see Abbreviations. Data represent the mean values ± SD (*n* = 3). Values in each column labeled with different capital letters (A–H) are significantly different at *p* < 0.05.

**Table 2:** Scavenging activity (SC_50_) of *P. spinosa* fruit extracts towards multiple oxidants.

	NO^•^ (µg/mL)	HOCl (µg/mL)	O_2_^•−^ (µg/mL)	H_2_O_2_ (mg/mL)	HO^•^ (mg/mL)
**Analytes:**					
MEF	18.28 ± 0.42 ^H^	40.04 ± 0.51 ^F^	73.89 ± 3.58 ^D^	0.17 ± 0.004 ^E^	0.44 ± 0.02 ^E,F^
MED	43.05 ± 3.08 ^I^	100.90 ± 0.69 ^G^	101.81 ± 3.05 ^E^	0.57 ± 0.007 ^J^	0.81 ± 0.02 ^H^
DEFF	5.61 ± 0.13 ^F^	**17.34 ± 0.50 ^B,C^**	**29.40 ± 0.88 ^B^**	**0.13 ± 0.002 ^C^**	**0.33 ± 0.01 ^D^**
DEFD	3.85 ± 0.22 ^D^	**18.35 ± 0.52 ^B,C^**	**32.70 ± 2.74 ^B^**	0.15 ± 0.002 ^D^	**0.32 ± 0.01 ^D^**
EAFF	**2.15 ± 0.16 ^C^**	31.48 ± 0.64 ^D^	45.08 ± 2.96 ^C^	**0.14 ± 0.001 ^C^**	**0.38 ± 0.01 ^D,E^**
EAFD	4.79 ± 0.39 ^E^	34.44 ± 0.46 ^D,E^	52.76 ± 2.82 ^C^	0.21 ± 0.005 ^F^	**0.33 ± 0.01 ^D^**
BFF	6.35 ± 0.41 ^G^	39.78 ± 0.85 ^F^	73.52 ± 2.68 ^D^	0.22 ± 0.002 ^G^	0.47 ± 0.01 ^F,G^
BFD	43.70 ± 2.49 ^I^	96.48 ± 5.41 ^G^	106.88 ± 4.39 ^E^	0.44 ± 0.008 ^I^	0.96 ± 0.06 ^I^
WRF	51.76 ± 3.89 ^J^	38.02 ± 0.88 ^E,F^	103.47 ± 4.06 ^E^	0.27 ± 0.002 ^H^	0.52 ± 0.03 ^G^
WRD	40.21 ± 1.99 ^I^	153.46 ± 0.83 ^H^	311.63 ± 7.08 ^G^	0.72 ± 0.003 ^K^	1.17 ± 0.06 ^J^
Quercetin	0.48 ± 0.03 ^A^	2.08 ± 0.14 ^A^	7.89 ± 0.24 ^A^	0.008 ± 0.001 ^A^	0.05 ± 0.002 ^A^
Cyanidin 3-*O*-glucoside	1.40 ± 0.09 ^B^	5.62 ± 0.11 ^A^	10.11 ± 0.33 ^A^	0.012 ± 0.001 ^A,B^	0.17 ± 0.002 ^C^
Chlorogenic acid	0.69 ± 0.02 ^A^	13.82 ± 0.53 ^B^	4.58 ± 0.29 ^A^	0.018 ± 0.001 ^A,B^	0.07 ± 0.001 ^A,B^
5-Hydroxymethylfurfural (HMF)	**	**	**	***	***
**Reference standards:**					
Ascorbic acid	0.59 ± 0.04 ^A^	6.32 ± 0.07 ^A^	5.28 ± 0.25 ^A^	0.014 ± 0.001 ^A,B^	0.14 ± 0.005 ^B,C^
Trolox	0.59 ± 0.02 ^A^	21.49 ± 1.21 ^C^	135.33 ± 5.59 ^F^	0.020 ± 0.001 ^B^	0.13 ± 0.003 ^B,C^

SC_50_, Half scavenging concentration (amount required to reduce the initial oxidant concentration by 50%) calculated in µg/mg of the dry extract or standard/mL of the reaction mixture; ** inactive up to a concentration of 500 µg/mL; *** inactive up to 1500 µg/mL. For extracts codes, see Abbreviations. Data are given as mean of three replicates ± SD. Values in each column labeled with different capital letters (A–K) are significantly different at *p* < 0.05.

## Data Availability

Data is contained within the article and supplementary materials.

## Supplementary Materials

The following supporting information can be downloaded at: https://www.mdpi.com/article/10.3390/ph15101300/s1, S1.: General; Table S1. Overall quantitative profile of the *P. spinosa* fruit extracts (mg/g dw); Table S2. Correlation (*r*) coefficients and probability (*p*) values of linear relationships between antioxidant and antidiabetic activity parameters and phenolic contents of *P. spinosa* fruits extracts. Table S3. Scavenging activity (SC_50_, µg GAE/mL) of *P. spinosa* fruit extracts towards multiple oxidants; Figure S1. Inhibitory capacity of *P. spinosa* fruit extracts, their activity markers and standards against glycolytic enzymes and protein glycation; Figure S2. Antioxidant activity of *P. spinosa* fruit extracts, their activity markers and standards towards in vivo-relevant oxidants.

## Abbreviations

3-NT-Fg	nitro-fibrinogen
ABTS	2,2′-azino-bis(3-ethylbenzothiazoline-6-sulfonic acid)
AGEs	advanced glycation end products
BFD	*n*-butanol fraction of MED
BFF	*n*-butanol fraction of MEF
DEFD	diethyl ether fraction of MED
DEFF	diethyl ether fraction of MEF
DM	diabetes mellitus
DPPH	2,2-diphenyl-1-picrylhydrazyl
dw	dry weight
EAFD	ethyl acetate fraction of MED
EAFF	ethyl acetate fraction of MEF
FRAP	ferric-reducing ability of plasma
GAE	gallic acid equivalents
H_2_O_2_	hydrogen peroxide
HMF	5-hydroxymetylfurfural
HO^•^	hydroxyl radical
HOCl	hypochlorous acid
MED	methanol–water (75:25, *v*/*v*) extract of dried fruits
MEF	methanol–water (75:25, *v*/*v*) extract of fresh fruits
MRPs	Maillard reaction products
NEAC	non-enzymatic antioxidant capacity of human plasma
NF-κB	nuclear factor kappa B
NO^•^	nitrogen oxide
O_2_^•−^	superoxide anion radical
ONOO^−^	peroxynitrite
PB2	procyanidin B2
RAGE	AGEs-specific receptor
RNS	reactive nitrogen species
ROS	reactive oxygen species
TAC	total content of anthocyanins
TBARS	thiobarbituric acid-reactive substances
TFL	total content of flavonoids
TPA	total content of phenolic acids
TPC	total phenolic content (Folin–Ciocalteu assay)
TPH	total phenolic content (sum of individual phenolics by HPLC)
TTC	total content of tannin-type proanthocyanidins
WRD	water residue of MED after fractionation
WRF	water residue of MEF after fractionation
