# Supplementary material for: Valorisation of the Inhibitory Potential of Fresh and Dried Fruit Extracts of Prunus spinosa L. towards Carbohydrate Hydrolysing Enzymes, Protein Glycation, Multiple Oxidants and Oxidative Stress-Induced Changes in Human Plasma Constituents
Source: Pharmaceuticals (Basel). 2022 Oct 21;15(10):1300. doi: 10.3390/ph15101300 (PMC9610855; doi:10.3390/ph15101300)
Supplement: Supplementary file 1 [file pharmaceuticals-15-01300-s001.zip › pharmaceuticals-1927404-supplementary materilas.pdf]

pharmaceuticals

MDPI

Supplementary Material for:

Valorisation of the Inhibitory Potential of Fresh and Dried Fruit Extracts of *Prunus spinosa* L. towards Carbohydrate Hydrolysing Enzymes, Protein Glycation, Multiple Oxidants and Oxidative Stress-Induced Changes in Human Plasma Constituents

Anna Magiera [1,*], Joanna Kołodziejczyk-Czepas [2], Karolina Skrobacz [2], Monika Ewa Czerwińska [3,4], Magdalena Rutkowska [1], Aleksandra Prokop [5], Piotr Michel [1] and Monika Anna Olszewska [1]

[1] Department of Pharmacognosy, Faculty of Pharmacy, Medical University of Lodz, 1 Muszynskiego St., 90-151 Lodz, Poland
[2] Department of General Biochemistry, Faculty of Biology and Environmental Protection, University of Lodz, 141/143 Pomorska, 90-236 Lodz, Poland
[3] Department of Biochemistry and Pharmacogenomics, Medical University of Warsaw, 1 Banacha St., 02-097 Warsaw, Poland
[4] Centre for Preclinical Research, Medical University of Warsaw, 1B Banacha St., 02-097 Warsaw, Poland
[5] Student Scientific Association, Department of Pharmacognosy, Faculty of Pharmacy, Medical University of Lodz, 1 Muszynskiego St., 90-151 Lodz, Poland
* Correspondence: anna.magiera@umed.lodz.pl; Tel.: +48 503316997

Table of Contents

S.1. General

Table S1. Overall quantitative profile of the *P. spinosa* fruit extracts (mg/g dw).

Table S2. Correlation (r) coefficients and probability (*p*) values of linear relationships between antioxidant and antidiabetic activity parameters and phenolic contents of *P. spinosa* fruits extracts.

Table S3. Scavenging activity (SC_{50}, μg GAE/mL) of *P. spinosa* fruit extracts towards multiple oxidants.

Figure S1. Inhibitory capacity of *P. spinosa* fruit extracts, their activity markers and standards against glycolytic enzymes and protein glycation.

Figure S2. Antioxidant activity of *P. spinosa* fruit extracts, their activity markers and standards towards *in vivo*-relevant oxidants.

S.1. General

High-purity reagents and standards for spectrophotometric and fluorometric assays: *α*-glucosidase; acarbose; aminoguanidine; fructose; sodium aside; *p*-nitrophenyl-*α*-D-glucopyranoside (*p*-NPG); bovine serum albumin (BSA); pancreatin from porcine pancreas; xanthine oxidase from bovine milk; xanthine; nitrotetrazolium blue chloride (NBT); hydrogen peroxide (H_2O_2); horseradish peroxidase; phenol; 4-aminoantipyrine; salicylic acid; iron (II) sulphate heptahydrate; 4,5-diaminofluorescein (DAF-2); 5-thio-2-nitrobenzoic acid (TNB); sodium borohydride; ethylenediaminetetraacetic acid; Evans blue; diethylenetriaminepentaacetic acid; Trolox; quercetin; isoquercitrin (quercetin 3-*O*-*β*-D-glucopyranoside); 5-hydroxymethylfurfural (HMF); chlorogenic acid (5-*O*-caffeoylquinic acid); and ascorbic acid; were purchased from Sigma-Aldrich (St. Louis, MO, USA), while sodium nitroprusside and sodium hypochlorite were obtained from Avantor Performance Materials (Gliwice, Poland); cyanidin 3-*O*-*β*-D-glucopyranoside (CYG) from Phytolab (Vestenbergsgreuth, Germany); and phosphate-buffered saline (PBS) from Biomed (Lublin, Poland). The EnzChek™ Ultra Amylase Assay Kit was purchased from Thermo Fisher Scientific (Walltham, USA). All immunoreagents for 3-nitrotyrosine detection were purchased from Abcam (Cambridge, UK). All other chemicals and solvents were of analytical grade and obtained from Avantor (Poland). In all analyses redistilled water was used. Samples were incubated in a constant temperature using a BD 23 incubator (Binder, Tuttlingen, Germany). All activity studies were performed using 96-well plates and monitored by microplate readers: SPECTROstar Nano (BMG LabTech, Ortenberg, Germany) or Synergy HTX (BioTek, Winooski, VT, USA).

Table S1. Overall quantitative profile of the *P. spinosa* fruit extracts (mg/g dw).

	TPC (GAE)	TPH	TPA	TAC	TFL	TTC (PB2)	MRPs
**Extracts/fractions::**							
MEF	87.57 ± 3.54^{C}	28.56 ± 0.58^{E}	19.67 ± 0.33^{G}	4.64 ± 0.11^{B}	4.25 ± 0.21^{E}	**44.53 ± 1.93^{A}**	n.d.
MED	26.77 ± 0.47^{F}	9.61 ± 0.60^{F}	8.19 ± 0.51^{H}	n.d.	1.43 ± 0.09^{F}	8.17 ± 0.24^{C}	0.92 ± 0.07^{C}
DEFF	**126.49 ± 1.41^{A}**	81.83 ± 0.80^{C}	35.15 ± 1.12^{D}	n.d.	**41.11 ± 0.41^{A}**	n.d.	n.d.
DEFD	**124.01 ± 0.70^{A}**	80.24 ± 1.16^{C}	47.51 ± 1.50^{C}	n.d.	25.99 ± 0.77^{B}	n.d.	**37.75 ± 2.39^{A}**
EAFF	**123.63 ± 3.68^{A}**	104.02 ± 1.92^{B}	91.26 ± 2.16^{B}	n.d.	12.21 ± 0.32^{C}	n.d.	n.d.
EAFD	107.43 ± 4.08^{B}	**109.91 ± 1.26^{A}**	**102.53 ± 0.85^{A}**	n.d.	6.69 ± 0.46^{D}	n.d.	13.53 ± 0.78^{B}
BFF	68.23 ± 0.12^{D}	43.17 ± 1.14^{D}	29.62 ± 1.10^{E}	**9.17 ± 0.33^{A}**	4.38 ± 0.17^{E}	8.02 ± 0.23^{C}	n.d.
BFD	46.58 ± 2.28^{E}	28.49 ± 0.96^{E}	25.16 ± 0.80^{F}	0.22 ± 0.01^{D}	3.12 ± 0.24^{E}	n.d.	1.37 ± 0.03^{C}
WRF	64.59 ± 0.61^{D}	6.07 ± 0.14^{G}	4.79 ± 0.08^{I}	0.96 ± 0.06^{C}	0.32 ± 0.02^{F}	28.36 ± 0.41^{B}	n.d.
WRD	22.59 ± 0.05^{F}	0.78 ± 0.01^{H}	0.78 ± 0.01^{J}	n.d.	n.d.	5.37 ± 0.11^{D}	0.26 ± 0.003^{C}

Results are presented as means ± SD (n = 3). For each parameter, different superscript letters indicate significant differences ($p <$ 0.05). The highest levels for each parameter are printed in bold. Extracts/fractions: MEF/MED, methanol-water (75:25, *v*/*v*) extracts of fresh/dried fruits; DEFF/DEFD, diethyl ether fraction of MEF/MED; EAFF/EAFD, ethyl acetate fraction of MEF/MED; BFF/BFD, *n*-butanol fraction of MEF/MED; WRF/WRD, water residue of MEF/MED. Analytical parameters: TPC, total phenolic contents in gallic acid equivalents (GAE); TPH, total contents of low molecular weight phenols determined by HPLC-PDA; TPA, total phenolic acids; TAC, total anthocyanins; TFL, total flavonoids; MRPs, total Maillard reaction products; TTC, total tannins in procyanidin B2 equivalents (PB2). N.d.: below the limits of quantitation (LOQ) or detection (LOD). For detailed quantitative levels and LC-MS/MS data for identification of individual compounds see the previous papers by Magiera et al. [1,2].

Table S2. Correlation (r) coefficients and probability (*p*) values of linear relationships between antioxidant and antidiabetic activity parameters and phenolic contents of *P. spinosa* fruits extracts.

r (*p*) for	α-glucosidase	α-amylase	AGEs fomation	NO	HOCl	$O_2^{\bullet-}$	H_2O_2	$HO^{\bullet}$
TPC	**-0.7273 (0.042)***	**-0.8328 (0.010)***	-0.4112 (0.312)	**-0.8814 (0.004)***	**-0.9282 (0.001)***	**-0.9636 (0.000)***	**-0.9153 (0.001)***	**-0.8873 (0.003)***
TPH	-0.3238 (0.434)	-0.5853 (0.127)	-0.0821 (0.847)	**-0.8082 (0.015)***	**-0.7408 (0.036)***	**-0.8281 (0.011)***	**-0.7096 (0.049)***	**-0.7529 (0.031)***
TFL	-0.6317 (0.093)	-0.5718 (0.139)	-0.1522 (0.719)	-0.5155 (0.191)	-0.6514 (0.080)	**-0.7991 (0.017)***	-0.5613 (0.148)	-0.5538 (0.154)
TAC	-0.1305 (0.758)	-0.1493 (0.724)	-0.6820 (0.062)	-0.1703 (0.687)	-0.1183 (0.780)	0.1925 (0.648)	-0.1702 (0.687)	-0.0899 (0.832)
TPA	-0.0425 (0.920)	-0.3624 (0.378)	0.0722 (0.865)	-0.6227 (0.099)	-0.4901 (0.218)	-0.5517 (0.156)	-0.4977 (0.209)	-0.5543 (0.154)
TTC	-0.3679 (0.370)	-0.1921 (0.649)	-0.5288 (0.178)	0.1141(0.788)	-0.0044 (0.992)	0.2332 (0.578)	-0.1042 (0.806)	-0.0407 (0.924)

Activity and concentration parameters according to Tables 1, 2, S1. Asterisk means statistical significance of the estimated linear relationships (**p* < 0 05). All statistically significant relationships are printed in bold.

Table S3. Scavenging activity (SC_{50}, μg GAE/mL) of *P. spinosa* fruit extracts towards multiple oxidants.

	$NO^{\bullet}$	HOCl	$O_2^{\bullet-}$	H_2O_2	$HO^{\bullet}$
**Extracts/fractions:**					
MEF	1.60	3.51	6.47	14.89	38.53
MED	1.15	2.70	2.73	15.25	21.68
DEFF	0.71	2.19	3.72	16.44	41.74
DEFD	0.48	2.28	4.06	18.60	39.68
EAFF	0.27	3.89	5.57	17.31	25.66
EAFD	0.51	3.70	5.67	22.56	35.45
BFF	0.43	2.71	5.02	15.01	32.07
BFD	2.04	4.49	4.98	20.49	44.72
WRF	3.34	2.46	6.68	17.44	34.05
WRD	0.91	3.47	7.04	16.26	26.43

SC_{50}, Scavenging efficiency (amount of antioxidant needed to decrease the initial concentration of the oxidant by 50%) expressed in μg of phenolics/mL of the enzyme reaction solution (values obtained by converting the original SC_{50} values using the TPC levels); expressed in μg of the dry extract or standard/mL of the reaction solution; GAE, gallic acid equivalents. For extracts/fractions codes, see Table S1 and Section Abbreviations. Results presented as mean values ± SD (n = 3).

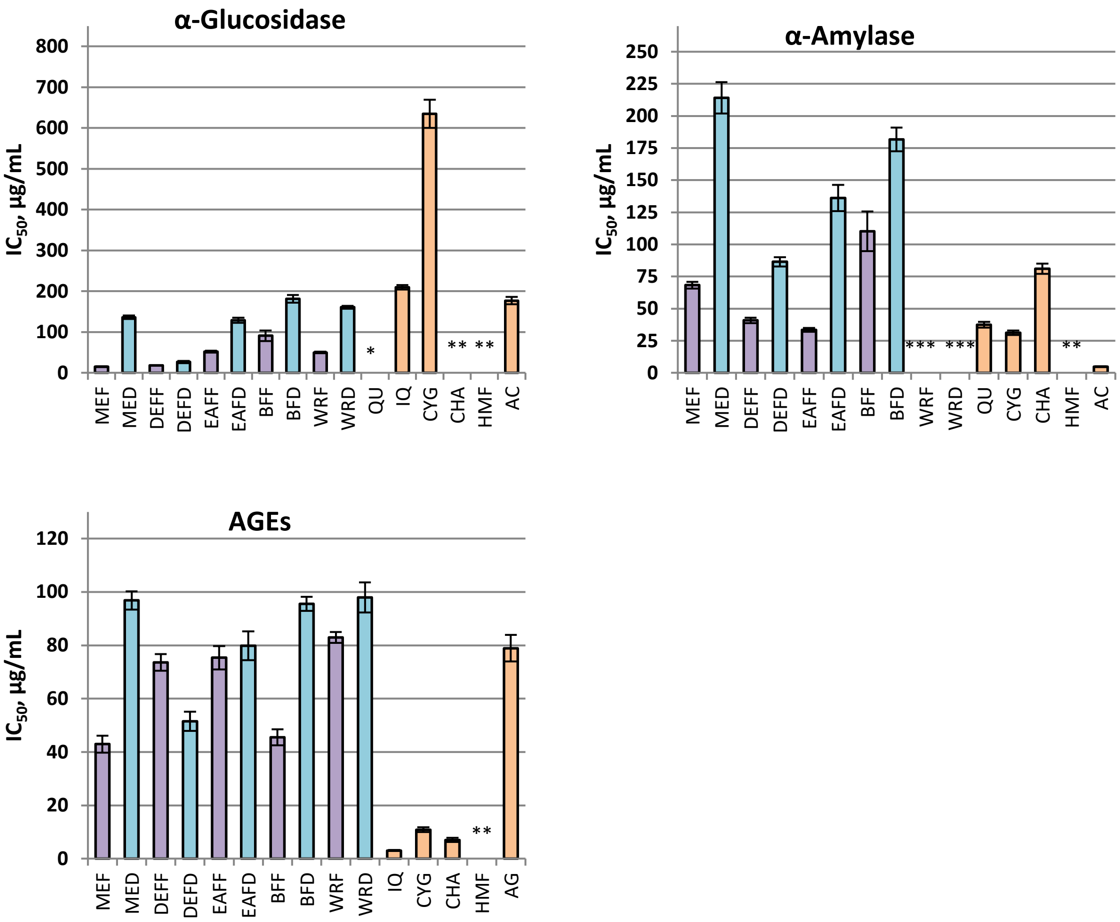

Figure S1. Inhibitory capacity of *P. spinosa* fruit extracts, their activity markers and standards against glycolytic enzymes and protein glycation. Results are presented as mean values of SC_{50} ± SD (n = 3), expressed in µg of the dry extract or standard/mL. Extracts/fractions: MEF/MED, methanol-water (75:25, *v*/*v*) extracts of fresh/dried fruits; DEFF/DEFD, diethyl ether fraction of MEF/MED; EAFF/EAFD, ethyl acetate fraction of MEF/MED; BFF/BFD, *n*-butanol fraction of MEF/MED; WRF/WRD, water residue of MEF/MED. QU, quercetin; IQ, isoquercitrin; CYG, cyanidin 3-*O*-glucoside; CHA, chlorogenic acid; HMF, 5-hydroxymethylfurfural; AC, acarbose; AG, aminoguanidine; * inactive up to a concentration of 50 µg/mL, above 50 µg/mL insoluble; ** inactive up to a concentration of 500 µg/mL; *** inactive up to 1500 µg/mL.

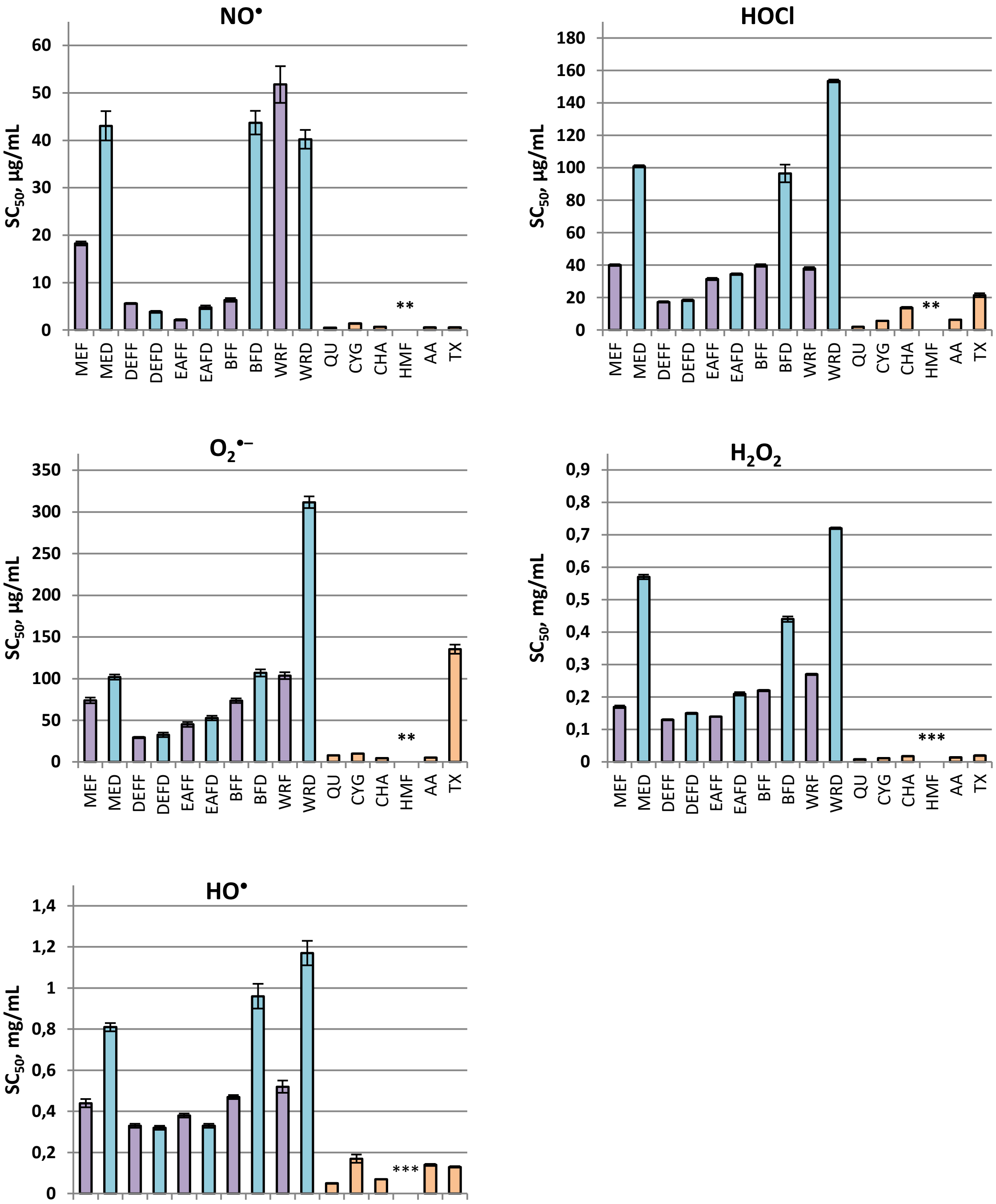

Figure S2. Antioxidant activity of *P. spinosa* fruit extracts, their activity markers and standards towards *in vivo*-relevant oxidants. Results are presented as mean values of SC_{50} ± SD (n = 3), expressed in µg/mg of the dry extract or standard/mL. Extracts/fractions: MEF/MED, methanol-water (75:25, *v*/*v*) extracts of fresh/dried fruits; DEFF/DEFD, diethyl ether fraction of MEF/MED; EAFF/EAFD, ethyl acetate fraction of MEF/MED; BFF/BFD, *n*-butanol fraction of MEF/MED; WRF/WRD, water residue of MEF/MED. QU, quercetin; CYG, cyanidin 3-*O*-glucoside; CHA, chlorogenic acid; HMF, 5-hydroxymethylfurfural; AA, ascorbic acid; TX, Trolox®; ** inactive up to a concentration of 500 µg/mL; *** inactive up to 1500 µg/mL.

References

1. Magiera, A.; Czerwińska, M.E.; Owczarek, A.; Marchelak, A.; Granica, S.; Olszewska, M.A. Polyphenol-enriched extracts of *Prunus spinosa* fruits: Anti-inflammatory and antioxidant effects in human immune cells ex vivo in relation to phytochemical profile. *Molecules* **2022a**, *27*, 1691.
2. Magiera, A.; Czerwińska, M.E.; Owczarek, A.; Marchelak, A.; Granica, S.; Olszewska, M.A. Polyphenols and Maillard reaction products in dried *Prunus spinosa* fruits: Quality aspects and contribution to anti-inflammatory and antioxidant activity in human immune cells ex vivo. *Molecules* **2022b**, *27*, 3302.
